# Interventions for Co-occurring Cannabis Use and Depression

**DOI:** 10.7759/cureus.27632

**Published:** 2022-08-03

**Authors:** Tomoya Sato

**Affiliations:** 1 Department of Plastic and Reconstructive Surgery, Saitama Medical University, Saitama, JPN; 2 Office of Public Health Studies, University of Hawaii, Honolulu, USA

**Keywords:** cannabis use, substance abuse, randomized controlled study, motivational interviewing, depression

## Abstract

This review aimed to investigate the effective intervention options for depression in patients with a history of cannabis use. The study eligibility criteria were as follows: English-language, peer-reviewed human studies; data not previously reported elsewhere; randomized controlled trials, non-randomized trials comparing an intervention group and a control group, and single-group trials.

In total, eight studies of interventions for patients with depression who reported cannabis use were identified. Four studies evaluated the effectiveness of the following three pharmacological interventions: extended quetiapine release, extended venlafaxine release, and fluoxetine. However, all studies failed to demonstrate the effectiveness of these drugs. Four studies evaluated the following psychological interventions: motivational interviewing (MI) and cognitive behavior therapy (CBT). These studies found that CBT may improve depression symptoms and cannabis dependence, and MI was associated with improvements in cannabis dependence.

CBT and MI may be effective in improving depression and reducing cannabis use. However, the conclusions of this review are limited because of the small number of studies and their low quality. Higher-quality research is required to evaluate the effectiveness of CBT, MI, and other interventions for comorbid cannabis use and depression.

## Introduction and background

Both depression and cannabis use are critical public health issues in the United States. In 2020, approximately 8.4% of adults had experienced one or more major depressive episodes (MDEs). This percentage has been increasing steadily over the last 10 years [1]. Depression is a significant health issue and a heavy burden on the healthcare system. Major depressive disorder (MDD) is the second most common cause of illness in terms of years lived with a disability [2]. The majority of mental disorders have their onset in adolescence [3] or during work years. Depression significantly decreases employee productivity and therefore affects not only public health but also the national economy.

Cannabis is the most commonly used illicit drug in the United States. According to the 2020 National Survey on Drug Use and Health (NSDUH), 49.6 million people in the United States aged 12 years and over used cannabis in the past year [1]. The peak period for substance use initiation was adolescence or the 20s. This is the period when the onset of depression is most likely to occur [4]. Cannabis can be used for several reasons, including coping with psychological distress, conformity to group norms, easing social interactions, and recreational purposes. Correlations were found between cannabis use and depression. More than 20% of adolescents who had experienced an MDE in the past year had also used cannabis in the past year compared to 7.9% of those without MDE. Additionally, about 40% of adults with severe mental disorders used cannabis in the past year, whereas 14.6% of those without severe mental disorders used cannabis [1].

Antidepressants and/or evidence-based psychotherapy are the first-line treatment for moderate and severe depression [5]. However, treating patients with depression using cannabis is less straightforward. Depression with comorbid substance abuse is correlated with a poorer prognosis, longer duration of treatment, and greater severity of symptoms [6]. Cannabis use has a significant negative effect on recovery from depression [7]. Cannabis use affects users’ mental status negatively and worsens depression, anxiety, suicide planning, and psychotic episodes [1]. Previous research has suggested that reducing cannabis use may improve the symptoms of depression [8]. The implementation of interventions for substance use and mental illness is complex, and interventions must be relevant to the target population [9]. Therefore, we cannot simply apply the guidelines for the treatment of depression in patients with co-occurring depression and cannabis use.

Recent studies have found that cannabis use, especially heavy use, is associated with an increased risk of developing depressive disorder [10]. Cannabis use in adolescents is strongly associated with depression, anxiety disorder, and suicidality in young adulthood [11]. Nunes and Levin [12] evaluated the effect of antidepressants on depression and drug dependence and found that antidepressants were effective in reducing the quantity of substance use, but not in abstinence. Thus, effective interventions for comorbid depression and cannabis use have not been identified, and further evaluation is needed.

This review aimed to investigate the efficacy of available intervention options for co-occurring depression and cannabis use.

## Review

Methodology

Study eligibility criteria were as follows: English-language, peer-reviewed human studies, and data not previously reported elsewhere. Study designs included were randomized controlled trials (RCTs), non-randomized trials, and single-group trials. The PubMed and PsycINFO bibliographic databases were used to identify relevant studies. A literature search was conducted in July 2022. No restrictions were placed on the publication date. The search consisted of two topics, depression and cannabis, using the following medical subject heading (MeSH) terms and keywords: (marijuana OR cannabis) AND (depression OR “depressive disorder”) AND (treatment OR intervention OR therapy).

Results

**Figure 1:**
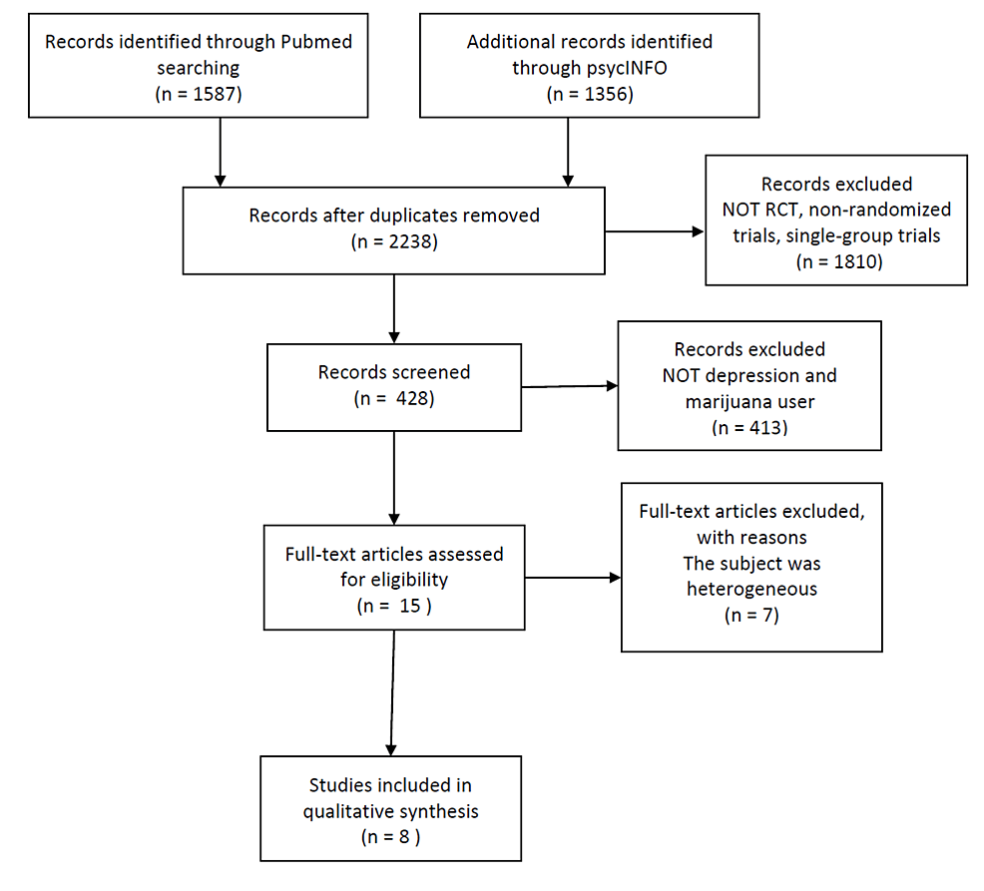
Flowchart of the study review process used in this review. RCT: randomized controlled trial

The literature searches identified 1,578 articles in PubMed and 1,356 articles in PsycINFO. A total of 2,238 articles were left after removing duplicates. Of these, 1,810 articles were not RCTs, non-randomized trials, or single-group trials and were removed from the list. Fifteen articles were considered relevant from the remaining 428. Seven items were removed because the subjects were heterogeneous, and the percentage of participants with depression and cannabis users was less than 20%. Eight studies were conducted among patients with depression who reported cannabis use: four articles on pharmacological intervention, two on cognitive behavior therapy (CBT), and two on motivational interviewing (MI) (Figure 1).

The characteristics of the studies included in this review are presented in Table 1.

**Table 1:** Summary of the characteristics of the studies for the intervention of co-occurring depression and substance use. RCT: randomized controlled trial; quetiapine-XR: extended quetiapine release; HDRS-17: Hamilton Depression Rating Scale-17; VEN-XR: extended venlafaxine release; HAM-D-27: Hamilton Rating Scale for Depression; BDI: Beck Depression Inventory; CDRS-R: Children’s Depression Rating Scale-Revised; CBT: cognitive behavior therapy; MDD: major depressive disorder; MI: motivational interviewing

Study	Number of patients	Age (years)	Design	Follow-up	Outcome	Summary estimate
Gao et al. 2017, USA [13]	90	18–65	RCT quetiapine-XR and placebo	8 weeks	HDRS-17	p = 0.41
					Cannabis use	p = 0.55
Levin et al. 2013, USA [14]	102	18–60	RCT VEN-XR and placebo	12 weeks	HDRS-17	X^2 ^= 0.48, p = 0.49
					Cannabis use	X^2 ^= 7.46, p < 0.01
Cornelius et al. 2010, USA [15]	70	14–25	RCT fluoxetine and placebo	12 weeks	HAM-D-27 BDI	p = 0.55, p = 0.80
					Cannabis use	p = 0.18
Findling et al. 2009, USA [16]	34	12–17	RCT fluoxetine and placebo	8 weeks	CDRS-R Cannabis use	p = 0.74, p = 0.64
Hides et al. 2009, Australia [17]	60	15–25	Single-group trial CBT	44 weeks	Full/partial remission of MDD	Week 20: 82.7% Week 44: 84.0%
					Cannabis use	Week 10: p = -0.006 Week 20: p = 0.010 Week 44: p = 0.007
Kay-Lambkin et al. 2009, Australia [18]	97	Over 16	RCT therapist delivered the CBT group (n = 35), the computer-delivered CBT group (n = 32), and the control group	12 months	BDI-II score	Odds ratio (95% CI)
					Therapist	2.29 (0.48, 11.00)
					Computer	3.89 (0.82, 18.39)
					Cannabis use	
					Therapist	2.00 (0.30, 13.51)
					Computer	4.69 (0.70, 31.21)
Satre et al. 2013, USA [19]	102	18 and over	RCT MI	6 months	Cannabis use	Effect size h
					3 months	0.61
					6 months	0.23
					BDI-II score	
					3 months	0.04
					6 months	0.06
Satre et al. 2016, USA [20]	307	18 and over	RCT MI	6 months	Cannabis use at 6 months	Effect size h, 0.23

Pharmacological Interventions

The effectiveness of pharmacological interventions was evaluated in four studies using three different drugs: venlafaxine-extended release (VEN-XR), quetiapine-extended release (quetiapine-XR), and fluoxetine. Each trial failed to demonstrate the effectiveness of these drugs for depression and cannabis use.

Gao et al. [13] conducted a randomized, double-blinded, placebo-controlled, eight-week study of quetiapine-XR. The study enrolled 90 patients. Of these, 46 patients were allocated to the quetiapine-XR group and 44 to the placebo group. Of the 46 patients, 22 had current alcohol or cannabis use disorder (ALC/CAN), and 24 did not. Of the 44 patients in the placebo group, 21 had current ALC/CAN, and 23 did not. The primary outcome measure was the improvement in depression, quantified by the difference from baseline to the end of the study using the Hamilton Depression Rating Scale-17 (HDRS-17). The secondary outcome measures were improvements on the Hamilton Anxiety Rating Scale, the 16-item Quick Inventory of Depressive Symptomatology-Self Report (QIDS-SR-16), the Clinical Global Impression for Bipolar Disorder-Severity, and the number of cannabis smoking days per week. There were no significant differences in HDRS-17 score as a result of treatment between the quetiapine-XR group and the placebo group. Among the secondary outcome measures, only the difference in changes in the QIDS-SR-16 scores reached significance. The main disadvantage of this study was the small number of participants and the fact that the participants had alcohol use disorder and cannabis use disorder. Larger studies are warranted to confirm the effectiveness of quetiapine-XR on depressive symptoms in patients with cannabis use disorders.

Levin et al. [14] compared VEN-XR with placebo. The primary outcome measures were abstinence from cannabis and improvement in depressive symptoms based on the HDRS-17. There were 51 patients in the VEN-XR and placebo groups. There was no significant difference in the improvement of depressive symptoms. The percentage of patients who achieved cannabis abstinence was significantly lower in the VEN-XR group than in the placebo group.

Two RCTs have examined the efficacy of fluoxetine for young individuals with co-occurring depression and cannabis use. Cornelius et al. [15] conducted an RCT of fluoxetine in adolescents and young adults (aged 14 to 25) with comorbid MDD and cannabis use disorder. All participants were initially given one capsule (10 mg fluoxetine or placebo) for two weeks. Then the dose was increased to two capsules (20 mg fluoxetine or placebo) from the third week. All of the participants in both groups had manual-based CBT and motivation enhancement therapy (MET) during the 12 weeks of the trial. Depressive symptoms were assessed with Hamilton Rating Scale for Depression (HAM-D-27) and the Beck Depression Inventory (BDI). There was no significant difference between the treatment group and placebo group in depression-related outcome variables and days of cannabis use during the past week at week 12. However, participants in both groups showed significant within-group improvement in depressive symptoms and cannabis use. The authors concluded that the improvement might result from CBT/MET psychotherapy’s efficacy or limited sample size.

Additionally, Findling et al. [16] conducted an eight-week RCT of fluoxetine among 34 depressed adolescents (aged 12 to 17) with alcohol (38.2%) and cannabis use disorders (88.2%). Participants received one capsule (10 mg fluoxetine or placebo) for the first four weeks. The dose could be increased to a maximum of 20 mg of fluoxetine or a matching placebo based on the physician’s discretion. The primary outcome was Children’s Depression Rating Scale-Revised (CDRS-R) scores and the percentage of patients with positive urine drug tests. There was no significant difference in the mean change in CDRS-R score (p = 0.74) and rates of positive urine drug toxicology results (p = 0.64) between the treatment group and the placebo group.

Psychological Interventions

Hides et al. [17] conducted a single-group study to evaluate the effectiveness of CBT for 60 young people (aged 15 to 25) with co-occurring depression (MDD) and substance misuse. In total, 32 (53.3%) participants had cannabis use disorder. The participants were provided a 20-week integrated CBT treatment and reassessed at mid-treatment (10 weeks), post-treatment (20 weeks), and six-month follow-ups (44 weeks) by research assistants. The primary outcome variables were depression and substance disorder. The measures of depression were the percentage of partial or full remission of MDD and the Hamilton Depression Rating Scale (HAM-D). At 20 weeks after CBT, 82.7% (n = 43) of the participants had full or partial remission of MDD. The number increased to 84.0% at six-month follow-ups. HAM-D mean scores at 10, 20, and 44 weeks were significantly lower than baseline scores (p < 0.001). The amount of cannabis use (g) also decreased significantly at week 10 (p = 0.006), week 20 (p = 0.010), and week 44 (p = 0.007). However, the study has limitations, including a small sample size, the lack of a comparison group, and no randomization.

Kay-Lambkin et al. [18] conducted an RCT to evaluate computer-based or therapist-delivered MI and CBT (intensive MI/CBT) for patients with comorbid major depression and alcohol and/or cannabis use at harmful levels. Participants were randomly allocated to the following three groups: a therapist-delivered CBT group (n = 35), a computer-delivered CBT group (n = 32), and a control group (n = 30). The primary outcome measures were depression symptoms quantified using the BDI-II and cannabis use. Twelve months after the intervention, a BDI-II score of <17 and/or a reduction in cannabis use of ≥50% were defined as improvements. Both the therapist-delivered and computer-delivered intensive MI/CBT groups showed improved depressive symptoms and cannabis dependence compared with the control group, but the improvements were not statistically significant.

MI was further tested in two different trials. Satre et al. [19] compared the MI (n = 52) and control (n = 52) groups. No significant differences were found in cannabis use reduction or improvement of depressive symptoms. Another RCT was conducted in 2016 by the same researchers [20]. They enrolled 307 patients and allocated 153 and 154 patients to the MI and control groups, respectively. The primary outcome measures were depression symptoms evaluated using the Patient Health Questionnaire (PHQ-9) depression scores and the percentage of patients who reported cannabis use. The PHQ-9 depression scores improved in both groups, but the difference was not statistically significant. The number of patients who had recently used cannabis was significantly lower in the MI group than in the control group (effect size h = 0.23). Logistic regression analysis showed that MI intervention was a negative predictor of cannabis use (odds ratio = 0.29).

Discussion

We conducted a literature review of interventions for comorbid cannabis use and depression. Eight studies were included in this review: four pharmacological treatment studies and four psychological treatment studies. None of the three drugs (quetiapine-XR, VEN-XR, and fluoxetine) showed an improvement in depressive symptoms or cannabis dependence. It is possible that the results did not reach statistical significance because the sample size in each study was small. Four studies on psychological interventions suggested that CBT and MI could improve depressive symptoms and cannabis dependence; however, further studies are needed to reinforce this conclusion.

Statistically, cannabis users are more likely to be diagnosed with depression than non-users. There is limited evidence that cannabis directly causes depression; however, recent research has shown that there are some genetic and neurological factors associated with comorbidity of cannabis use and depression. Cannabis use causes neurological change, particularly in the dorsal striatum, and the neuroplastic change is a possible mechanism resulting in the inability to feel pleasure and lack of motivation [21].

Based on this review, no conclusion can be drawn regarding the effectiveness of pharmacological interventions for comorbid cannabis use and depression. There is insufficient evidence to support the efficacy of psychological interventions for the reduction of symptoms and severity of depression. Two studies [15,17] showed that CBT might be effective in improving depressive symptoms and reducing the amount of cannabis use. The conclusion was limited because of the small sample size and the lack of a comparison group. Further studies using randomized controlled designs are needed to test the efficacy of CBT. We also found some evidence to support the efficacy of MI in reducing cannabis use, as measured six months after the intervention, but the effect was small and inconsistent. Recent guidelines on cannabis use disorder in adults also recommend MI and other psychological approaches for harm reduction and relapse prevention [22]. MI can be considered for patients with coexisting cannabis use and depression; however, the positive effects are minimal and may not be seen immediately.

This study had several limitations. We were able to identify seven relevant RCTs and one single-group trial. Most studies in this review had heterogeneous participants with multiple mental disorders and several types of substance users. The sample sizes of the included studies were small and therefore less likely to be representative of the target population. The methods of statistical analysis varied among the studies, preventing a meta-analysis.

Further, higher-quality experimental studies are needed in this area to investigate effective interventions. Pharmacological interventions are the cornerstones of the treatment of depression. More than 20 drugs [23] and numerous psychological interventions [24], including CBT, interpersonal therapy, and behavioral activation, are recommended in the treatment guidelines for depression. However, only three drugs have been evaluated by RCTs for their effectiveness in the treatment of co-occurring cannabis use and depression. The efficacy of these interventions should be evaluated in future research.

There is also a need for trials with homogeneous populations. The heterogeneity of patient populations can pose a challenge in human medical trials. Mental disorders and substance abuse are intricately intertwined. A large percentage of cannabis users tend to use other substances [25], and many individuals with depression have other concurrent mental disorders [26]. Trials with populations that specifically present with comorbid cannabis use and depression are required.

## Conclusions

We conducted a review of studies on interventions for co-occurring cannabis use and depression. CBT may improve depression symptoms and cannabis dependence, and MI may be effective in reducing cannabis use for at least six months after the intervention. However, the conclusions of this review are limited owing to the small number and low quality of the included studies. More high-quality research is required to evaluate the effectiveness of CBT and MI and other interventions on comorbid depression and cannabis use.
